# Astaxanthin Alleviates Ochratoxin A (OTA)-Induced Spleen Dysfunction and Apoptosis in Broiler Chickens by Modulating the PTEN/PI3K/AKT Signaling Pathway

**DOI:** 10.3390/antiox14101160

**Published:** 2025-09-24

**Authors:** Zhibi Cheng, Weilun Sang, Peng Li, Shuhua Yang

**Affiliations:** College of Animal Science and Veterinary Medicine, Shenyang Agricultural University, Shenyang 110866, China; 2023240780@stu.syau.edu.cn (Z.C.); swl524@stu.syau.edu.cn (W.S.)

**Keywords:** ochratoxin (OTA), apoptosis, astaxanthin, immunotoxicity, oxidative stress injury, chicken

## Abstract

Ochratoxin A (OTA), a common mycotoxin contaminant, poses significant health risks through its multi-organ toxicity. While OTA is known to cause immune organ dysfunction leading to immunotoxicity, its precise mechanistic pathways remain unclear. The spleen is an important immune organ of the body and plays a key role in immune defense and homeostasis maintenance. Astaxanthin (AST), a potent antioxidant with demonstrated immunomodulatory properties, exhibits a broad therapeutic potential including anti-inflammatory, wound-healing, anti-aging, and hepatoprotective effects. Therefore, this study aimed to explore the mechanism by which AST attenuates OTA-induced immunotoxicity using a chicken OTA/AST treatment model. Sixty 1-day-old, white-feathered, sex-undifferentiated chicks were randomly allocated into four groups (n = 15): (1) Control, (2) OTA (1 mg/kg), (3) AST (100 mg/kg), and (4) OTA + AST (1 mg/kg OTA + 100 mg/kg AST). The experiment lasted for 21 days to establish the model. Subsequently, serum ELISA, antioxidant capacity assays, qRT-PCR, and western blot (WB) analyses were employed to explore the protective role of AST against immunotoxicity. The results showed that AST increased splenic organ coefficients and serum immunoglobulin (IgM and IgG) concentrations (*p* < 0.01) and decreased the expression of inflammatory factors (IL-8, IL-6, and IL-1β) (*p* < 0.01). We found that OTA was involved in the expression of the PTEN/PI3K/AKT signaling pathway (PTEN, PI3K, AKT, p-AKT (Ser473)) and apoptotic genes (Bcl-2, Bax, Caspase3, Caspase9). Notably, AST significantly attenuated OTA-induced oxidative damage (ROS, MDA, T-AOC) in the spleen (*p* < 0.05), upregulated the expression of PI3K and p-AKT (Ser473) (*p* < 0.05) and inhibited the expression of PTEN and apoptosis-related genes (*p* < 0.05). In summary, AST attenuates OTA-induced immunotoxicity by alleviating oxidative stress and modulating the PTEN/PI3K/AKT signaling pathway.

## 1. Introduction

Ochratoxin, a major metabolite derived from toxigenic fungi, stands as the predominant natural contaminant in both human food and animal feed [1]. It exists in four distinct forms: A, B, C, and D. Among the four types, OTA exhibits the highest toxicity [2,3]. As the most toxic and pervasive contaminant in both animal feed and human food, it has been categorized as a Group 2B possible human carcinogen by the International Agency for Research on Cancer (IARC) [4,5]. OTA can cause a variety of toxic effects in animals, including hepatotoxicity, nephrotoxicity, immunotoxicity, and genotoxicity [6,7,8,9]. The immune system is highly sensitive to OTA [10,11], and its toxicity is characterized by reducing the size of immune organs in each species [12]. Even low concentrations of OTA reduce the size of immune organs and decrease serum globulin (IgA, IgG, IgM) concentrations [13]. The immunotoxicity of OTA was found to be associated with oxidative stress. OTA induces oxidative stress in chicken immune organs, decreases the activities of CAT and GSH and increases MDA accumulation [14]. OTA increases ROS levels, apoptosis, and LDH release, decreases cell viability, and induces oxidative stress in porcine alveolar macrophages [15].

The PTEN/PI3K/AKT pathway is closely associated with apoptosis. PTEN is a protein that, when increased, negatively regulates the PI3K/AKT signaling pathway, and oxidative stress can inhibit the expression of the PI3K/AKT pathway by producing PTEN [16]. The PI3K/AKT pathway is responsible for normal proliferation, metallogenesis, growth, and cell survival in response to different stimuli, and it can regulate a variety of downstream substrates such as the apoptosis-related protein Caspase9 [17]. PI3K (phosphatidylinositol kinase) can regulate the binding of subunit p85 to the p110 catalytic subunit, constituting a heterodimer of PI3K protein [18]. As a downstream protein of PI3K, AKT mainly has two vital phosphorylation sites: Thr308 and Ser473 [19]. Anti-cancer drugs (e.g., paclitaxel and rottlerin) have been found to restrain cell proliferation and invasion and promote apoptosis by inhibiting p-AKT (Thr308) and p-AKT (Ser473) in different cancer cells [20,21]. It has been found that OTA can lead to apoptosis and necrosis through the PTEN/PI3K/AKT pathway [22]. However, it is not clear if OTA causes immunotoxicity in chickens through the PTEN/PI3K/AKT pathway.

Currently, the addition of antioxidants to polluted feeds is an effective way to reduce mycotoxin toxicity [23,24]. AST is an red lipophilic keto-carotenoid pigment found in certain algae [25], which has anti-inflammatory, anti-aging, immunomodulatory, and DNA repair properties [26]. The antioxidant property of AST is the absorption of free radicals into the polyene chain or the reaction with substances to form chemical bonds [27]. This antioxidant activity is 500 times greater than vitamin E and has 38 times the potential to terminate the free radical chain reaction than beta-carotene [28]. AST has been found to mitigate ochratoxin A-induced hepatotoxicity, nephrotoxicity, and cardiorespiratory dysfunction [29,30,31]. AST is used as a dietary additive. Supplementation of astaxanthin (40 or 80 mg/kg) in chicken diets can improve the meat quality, growth rate, and immunity of chickens under high temperature conditions. These reports suggest that astaxanthin is protective against OTA toxicity but is not known for immunotoxicity.

Through animal experiments, this study systematically elucidated the protective mechanism of Astaxanthin against ochratoxin A-induced immunotoxicity in chickens. It aimed to reveal how AST alleviates OTA-induced splenic damage by mitigating oxidative stress and modulating the PTEN/PI3K/AKT apoptotic pathway. To our knowledge, this is the first study to demonstrate the protective role of AST in regulating the PTEN/PI3K/AKT pathway against OTA-induced apoptosis and oxidative damage in avian splenocytes.

## 2. Materials and Methods

### 2.1. Specialized Feed Preparation

The *Aspergillus ochraceus AS3.3876* strain was obtained from the Guangdong Provincial Microbial Culture Collection Center (GDMCC, Guangzhou, China). The lyophilized fungus was first resuscitated by dissolving and inoculating it onto Luria–Bertani (LB) solid medium, followed by incubation for 2–3 days. Subsequently, feed was inoculated with a suspension of A. *Ochraceus conidia* and incubated at 29–30 °C for 14 days to promote OTA production. The OTA concentration in the resulting moldy feed was quantified using high-performance liquid chromatography with ultraviolet detection (HPLC-UV; Thermo Fisher Scientific, Waltham, MA, USA). Finally, following the protocol of Dhanshetty et al. [32], the moldy feed was thoroughly blended with normal feed in a specific ratio to achieve the desired OTA concentration for the experimental diet [33]. AST supplementation was administered as *Haematococcus pluvialis powder* (1.14% effective AST content; Yunnan Aier Occurrence Technology Co., Ltd., Chuxiong, China), a primary commercial natural AST source extracted from freshwater microalgae. AST-supplemented feed (100 mg/kg) was prepared according to the protocol of Hosseindoust et al. [34] by thoroughly mixing AST with normal feed in the appropriate proportion. The doses of OTA [35,36] and AST [29,37] were selected based on previous reports. Feed for all experimental groups was prepared simultaneously, portioned, and stored in resealable bags at 4 °C until use.

### 2.2. Animal Research

Sixty healthy 1-day-old chickens were obtained from a commercial farm (Shenyang Poultry Farm, Shenyang, China). Prior to use, all coops, cages, feeders, and drinkers in the facility were thoroughly cleaned, disinfected, and fumigated. The white-feathered chicks (mean weight: 45.365 g; 1 day old) were housed in three-tier cages under controlled conditions: 32 ± 5 °C and 40 ± 5% humidity. After transportation, the chickens were allowed to acclimate for 7 days before the experiment. They were randomly assigned to four groups: Control, AST (100 mg/kg AST), OTA (1 mg/kg OTA), and AST + OTA (1 mg/kg OTA + 100 mg/kg AST). Chicks had ad libitum access to feed and water for 21 days. From day 1 to 15, chickens were fed a standard starter pellet diet (Wellhope Foods Co., Ltd., Liaoning, China) containing 22.8% crude protein and 3000 kcal ME/kg. From day 16 to 28, they were switched to a grower ration (21.8% crude protein and 3150 kcal ME/kg) from the same manufacturer. All diets complied with food hygiene standards, met the nutritional requirements of the animals, and were free of medications. All animal procedures complied with the “Experimental Animal Management Regulations of the People’s Republic of China” and were approved by the Institutional Animal Care and Use Committee of Shenyang Agricultural University (Approval No. 201806014).

### 2.3. Collection of Samples

On day 21, the body weight of chickens was measured. Following blood sample collection via wing vein puncture, chickens were euthanized by cervical dislocation, and their spleens were harvested. Excess blood on the spleen surface was blotted dry with filter paper, and the precise weight was recorded. Subsequently, each spleen was dissected into two portions: one was fixed in 4% paraformaldehyde for H&E staining, while the other was placed in labeled cryostat tubes and stored at −80 °C until use.

spleen index = (spleen mass/body mass) × 100%

### 2.4. Histopathology

The excised spleen tissues were washed with saline and immediately placed in 4% paraformaldehyde fixative. Then, they were processed via gradient alcohol dehydration (50%, 70%, 80%, 100%), made transparent, dipped in wax, embedded, and sectioned (4–6 μm). Staining was carried out according to the instructions of the corresponding kit (Seville Biotechnology, Wuhan, China).

### 2.5. TUNEL Apoptosis Analysis

Samples were processed in accordance with the corresponding kit protocols. For TUNEL analysis, paraffin-embedded spleen sections were utilized (Liaoning Jijia Biotechnology, Wuhan, China).

### 2.6. Serum IgM and IgG Assays

ELISA kits for the detection of serum IgM and IgG levels in chicken were provided by JONLN Biotechnology (Shanghai, China).

### 2.7. Analysis of Splenic Oxidative Parameters

Kits for ROS, MDA, and T-AOC level analysis were provided by Nanjing Jiancheng Bioengineering Institute (Nanjing, China). To a weight of precisely 1 g of spleen tissue, add 9-fold saline, and prepare a 10% homogenate. After centrifugation at 3000× *g* for 15 min at 4 °C, carefully aspirate the supernatant and dilute it with saline to the optimal concentration. The homogenate supernatant was then assayed for ROS, MDA, and T-AOC levels.

### 2.8. RNA Extraction and Real-Time Fluorescence Quantitative PCR

Total mRNA was extracted using Trizol reagent (Vazyme, Nanjing, China). RNA purity and concentration were determined via absorbance ratios at 260/280 nm. cDNA was synthesized from 1 μg of total RNA using the Starscript III One-Step qRT-PCR SYBR kit (GenStar, Beijing, China). qRT-PCR analysis of β-actin, PTEN, PI3K, AKT, Bcl-2, Bax, Caspase3, IL-8, IL-6, and IL-1β was performed using the 2×RealStar Fast SYBR qPCR Mix kit (GenStar, Beijing, China) on an ABI iQ5 Fluorescence Quantitative PCR Detection System (ABI, Waltham, MA, USA). Sangon Biotech Co., Ltd. (Shanghai, China) designed and synthesized the primer pairs (Table 1). All data were normalized to β-actin and analyzed using the 2^−ΔΔCt^ method.

### 2.9. Western Blot Analysis

Protein expression of PTEN (1:500,Wanleibio, Shenyang, China), PI3K (1:2000, Wanleibio, Shenyang, China), p-AKT (1:200, Biodragon, Suzhou, China), AKT (1:1000, Wanleibio, Shenyang, China), Caspase3 (1:1000, proteintech, Wuhan, China), Caspase9 (1:800, proteintech, Wuhan, China), Bax (1:1000, Immunoway, Suzhou, China), Bcl-2 (1:1000, abmart, Shanghai, China), and GAPDH (1:10,000, epizyme, Shanghai, China) in the spleen of chickens was determined by WB. Proteins were extracted from spleen tissue using a total protein extraction kit (containing RIPA and PMSF; Yase, Shanghai, China). The total protein content in chicken spleen was quantified with a BCA protein assay kit (Nanjing Jiancheng Bioengineering Institute, Nanjing, China). Proteins were resolved by SDS-PAGE and transferred to a PVDF membrane (Yase, Beijing, China). Non-specific binding was minimized by incubating the membrane overnight at 4 °C with blocking buffer (5% skim milk). The PVDF membrane was then incubated overnight at 4 °C with primary antibody diluted in skim milk. Following six washes with TBST, the membrane was incubated for 60 min with horseradish peroxidase (HRP)-conjugated goat anti-rabbit IgG (1:20,000; Epizyme, Shanghai, China). Signal development was performed using an enhanced chemiluminescence (ECL) substrate (Beyotime Biotechnology, Shanghai, China). Band intensities were normalized to GAPDH as the loading control, and relative quantification was performed using the ImageJ 1.51 software.

### 2.10. Statistical Analysis of Data

Statistical analyses were conducted using the IBM SPSS Statistics 25 software (SPSS Inc., Chicago, IL, USA), with results expressed as mean ± standard deviation (mean ± SD). To compare differences among experimental groups (Control, AST, OTA, and AST + OTA), data normalization was first performed. Subsequently, one-way ANOVA followed by Tukey’s multiple comparison tests were applied. Graphs were generated using the GraphPad Prism 10 software (GraphPad Software, La Jolla, CA, USA). *p* < 0.05 was considered statistically significant, while *p* < 0.01 indicated a highly significant difference.

## 3. Results

### 3.1. Effect of AST on OTA-Induced Changes in the Spleen Index

**Figure 1:**
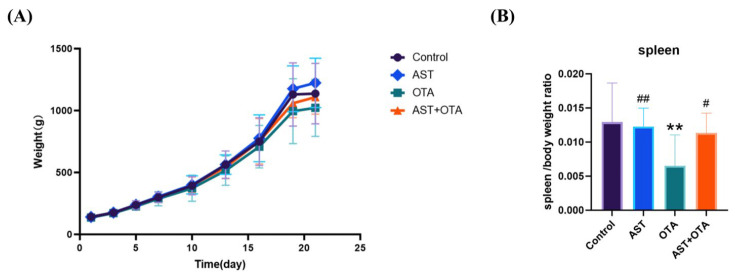
(**A**): Chicken weight growth changes. (**B**): Chicken spleen index ratio. Values represent mean ± SD. ** *p* < 0.01 vs. Control group; # *p* < 0.05 vs. OTA group; ## *p* < 0.01 vs. OTA group.

Body weight changes and the spleen organ index were recorded in chickens to evaluate OTA-induced spleen injury and the protective effect of AST. As shown in Figure 1A,B, no significant growth retardation occurred during 0–15 days, but from 15 to 21 days, the OTA group exhibited significantly lower average body weight and spleen index than the Control group (*p* < 0.01). Notably, the AST + OTA group showed an elevated splenic index compared with the OTA group (*p* < 0.05), suggesting that AST effectively mitigates OTA-induced immunotoxicity.

### 3.2. Histopathological Changes in the Spleen

**Figure 2:**
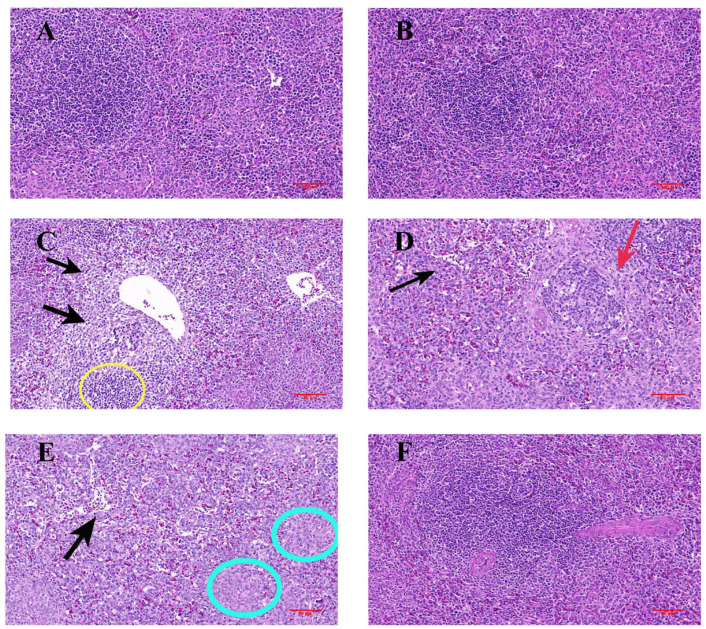
H&E staining of spleen in each group (400× magnification). (**A**) Control group, (**B**) AST group, (**C**–**E**) OTA group showing black arrows: splenic blood sinus dilatation; yellow circles: inflammatory cell infiltration; red arrows: sparingly distributed splenic nodules; blue circles: large necrotic foci. (**F**) AST + OTA group.

To assess OTA-induced immunotoxicity, we conducted the H&E staining of chicken spleen tissue. Figure 2A,B reveals a tight and regular arrangement of splenic red and white pulp in the Control and AST groups. In contrast, Figure 2C–E demonstrates that the OTA group exhibited poorly demarcated red and white pulp, broadly dilated and hemorrhagic splenic sinuses, extensive splenocyte vacuolation within nodules, and severe lymphocyte depletion. Figure 2F shows that the AST + OTA group markedly attenuated OTA-induced histological damage.

### 3.3. Analysis of Apoptosis by TUNEL

**Figure 3:**
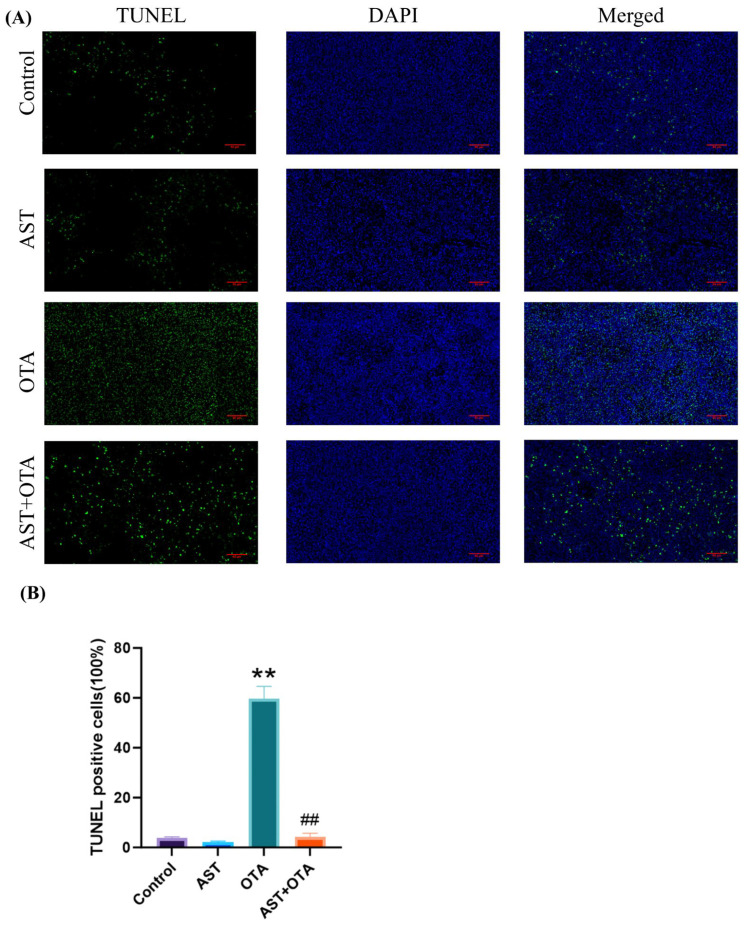
(**A**) TUNEL staining. Green fluorescence indicates TUNEL-positive cells. Blue fluorescence indicates DAPI staining of nuclei (magnification 400×). (**B**) TUNEL-positive cells (%). ** *p* < 0.01 vs. Control group; ## *p* < 0.01 vs. OTA group.

Figure 3A,B shows minimal green fluorescence in the Control and AST groups, indicating fewer apoptotic cells. The OTA group exhibited significantly stronger green fluorescence than the Control group *(p* < 0.01), demonstrating extensive apoptosis in the spleen. In contrast, the AST + OTA group showed markedly reduced fluorescence (*p* < 0.01), confirming that AST significantly inhibits OTA-induced apoptosis.

### 3.4. Effect of AST on OTA-Induced Serum Immunoglobulins

**Figure 4:**
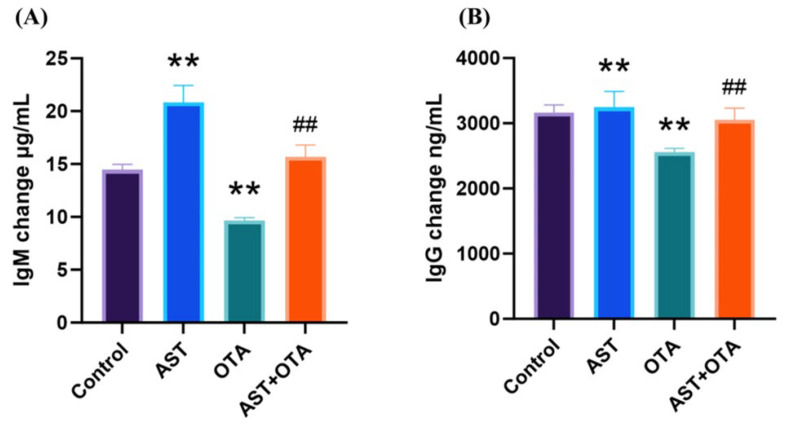
Effect of AST on immunoglobulins in serum of OTA-exposed chickens. (**A**) Serum IgM concentration levels in chickens; (**B**) Serum IgG concentration levels in chickens. ** *p* < 0.01 vs. Control group; ## *p* < 0.01 vs. OTA group.

To evaluate OTA’s effects on serum immunoglobulins and AST’s protective role, we measured serum IgM and IgG concentrations. Figure 4A shows that compared with the Control group, the OTA group exhibited significant decreases in both IgM and IgG levels (*p* < 0.05). Notably, the AST + OTA group demonstrated significantly higher immunoglobulin concentrations than the OTA group (*p* < 0.01). These findings demonstrate that AST effectively protects against OTA-induced immunoglobulin reduction.

### 3.5. AST Mitigates the Effects of OTA-Induced Oxidative Stress

**Figure 5:**
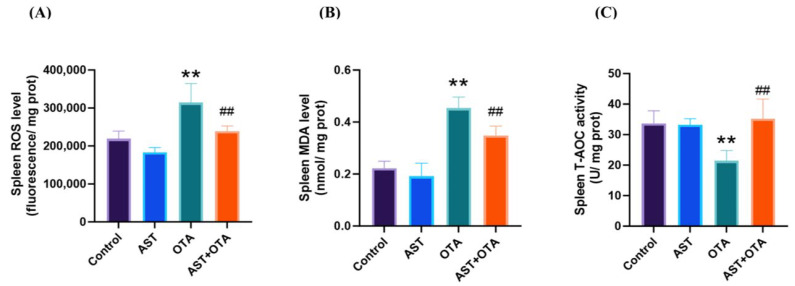
Effect of OTA on oxidative stress indices in chicken spleen. (**A**) ROS level; (**B**) MDA level; (**C**) T-AOC activity. ** *p* < 0.01 vs. Control group; ## *p* < 0.01 vs. OTA group.

Figure 5A,B demonstrates that splenic ROS and MDA levels were significantly elevated in the OTA group compared with the Control group (*p*< 0.01). In contrast, the AST + OTA group showed markedly reduced ROS and MDA levels relative to the OTA group (*p* < 0.01). As shown in Figure 5C, T-AOC activity was significantly lower in the OTA group than in the Control group (*p* < 0.05), while the AST + OTA group exhibited increased T-AOC activity compared with the OTA group (*p* < 0.05). These results indicate that OTA significantly increases ROS production and MDA accumulation while impairing antioxidant capacity in chicken spleen, whereas AST effectively mitigates OTA-induced oxidative stress.

### 3.6. PTEN/PI3K/AKT (Ser473) Pathway, Apoptosis and Inflammatory Factor-Related Gene Expression

**Figure 6:**
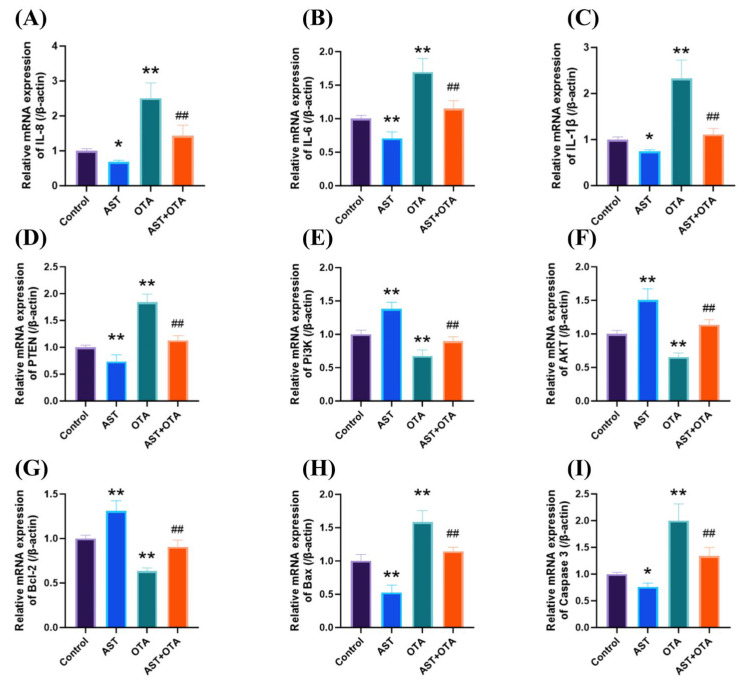
PTEN/PI3K/AKT (Ser473) pathway, apoptosis, and inflammatory factor-related gene expression. (**A**) IL-8, (**B**) IL-6, (**C**) IL-1β, (**D**) PTEN, (**E**) PI3K, (**F**) AKT, (**G**) Bcl-2, (**H**) Bax, (**I**) Caspase3. * *p* < 0.05 vs. Control group; ** *p* < 0.01 vs. Control group; ## *p* < 0.01 vs. OTA group.

To investigate the involvement of the PTEN/PI3K/AKT (Ser473) signaling pathway in AST-mediated protection against OTA-induced apoptosis and inflammation in chicken splenocytes, we quantified the mRNA expression levels of IL-8, IL-6,IL-1β,PTEN, PI3K, AKT, Bcl-2, Bax, and Caspase3. Figure 6 demonstrates that OTA exposure significantly upregulated IL-8, IL-6, IL-1β, PTEN, Bax, and Caspase3 mRNA expression (*p* < 0.01), while downregulating PI3K, AKT, and Bcl-2 expression compared with the Control group (*p* < 0.01). Compared with the OTA group, the relative expression levels of IL-8, IL-6, IL-1β, PTEN, Bax, and Caspase3 mRNA were downregulated in the AST + OTA group (*p* < 0.05), and the relative expression levels of PI3K, AKT, and Bcl-2 mRNA were upregulated in the AST + OTA group (*p* < 0.01). These findings strongly indicate that AST exerts protective effects against OTA-induced immunotoxicity through modulation of the PTEN/PI3K/AKT signaling pathway.

### 3.7. PTEN/PI3K/AKT (Ser473) Pathway and Apoptosis-Related Protein Expression

**Figure 7:**
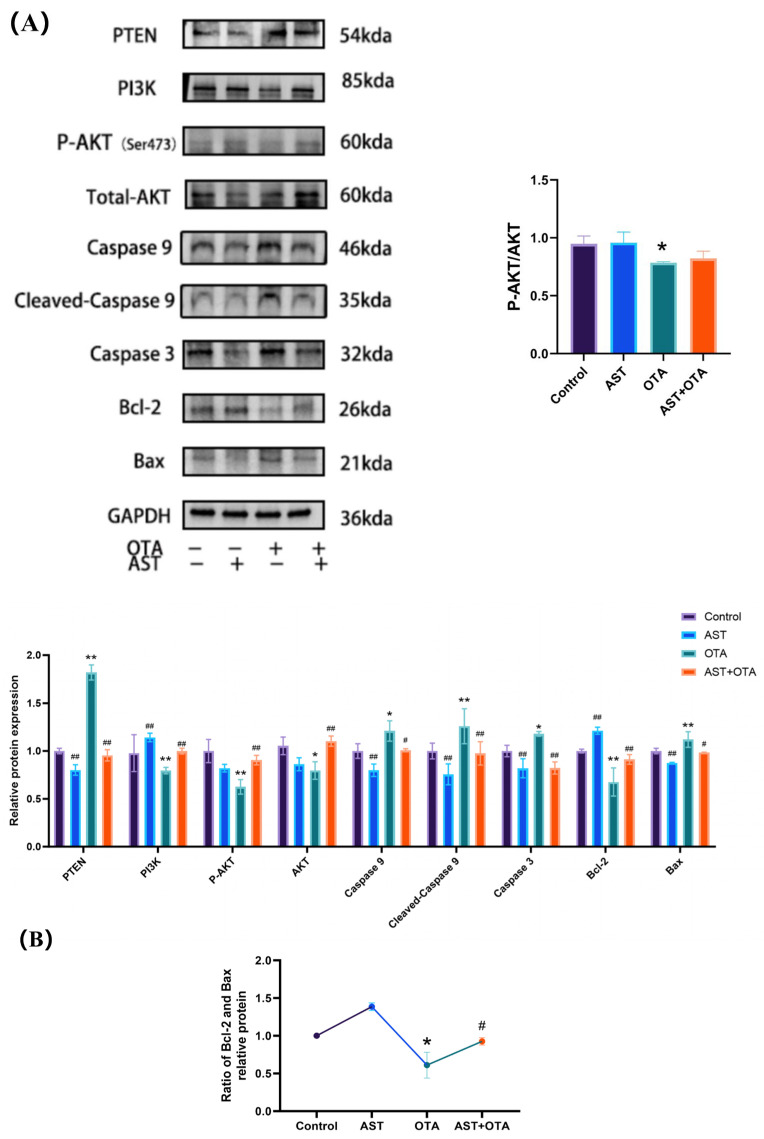
PTEN/PI3K/AKT pathway and apoptosis-related protein expression. (**A**) PTEN/PI3K/AKT pathway relative protein expression; (**B**) Ratio of Bcl-2 and Bax. * *p* < 0.05 vs. Control group; ** *p* < 0.01 vs. Control group; # *p* < 0.05 vs. OTA group; ## *p* < 0.01 vs. OTA group.

To elucidate the involvement of the PTEN/PI3K/AKT (Ser473) signaling pathway in AST’s protective effects against OTA-induced apoptosis in chicken splenocytes, we examined key protein expression levels. Figure 7A reveals that OTA exposure significantly downregulated PI3K, p-AKT (Ser473), AKT, and Bcl-2 protein expression (*p* < 0.05), while upregulating PTEN, Caspase9, cleaved Caspase9, Caspase3, and Bax (*p* < 0.05). The protein expression levels of PI3K, p-AKT (Ser473), AKT, and Bcl-2 were significantly upregulated (*p* < 0.05) in the AST + OTA group compared with the OTA group, while PTEN, Caspase9, cleaved Caspase9, Caspase3, and Bax showed significant downregulation (*p* < 0.05). As shown in Figure 7B, Bcl-2/Bax ratio decreased in the OTA group compared with the Control group (*p* < 0.05). The Bcl-2/Bax ratio increased in the AST + OTA group compared with the OTA group (*p* < 0.05). These results demonstrate that AST protects against OTA-induced splenocyte apoptosis through modulation of the PTEN/PI3K/AKT (Ser473) signaling pathway.

## 4. Discussion

In recent years, mycotoxin contamination of food crops has become a serious problem for animal husbandry. According to the Food and Agriculture Organization of the United Nations (FAO), over 25% of global food crops are contaminated with mycotoxins annually [38]. OTA is among the most prevalent mycotoxins, detected in various grains and animal-derived foods; grains contribute to approximately 60% of total OTA exposure. OTA contamination in animals reduces feed conversion efficiency, compromises health and welfare, and inflicts significant economic losses on production systems. Furthermore, due to its poor metabolizability, OTA accumulates in tissues such as meat, offal, and eggs, thereby posing a substantial threat to human health. Consequently, developing effective dietary interventions to mitigate OTA toxicity in livestock production is urgently needed. Poultry are particularly vulnerable to OTA toxicity [39,40]. In the present study, we established an OTA-induced chicken spleen injury model to systematically evaluate both the immunotoxic effects of OTA-contaminated feed and the potential protective efficacy of AST.

Previous studies have demonstrated that prolonged OTA exposure (400–800 μg/kg for 42 days) induces lymphocyte depletion and tissue damage in porcine spleens [41]. Similarly, chick exposure to 0.5 mg/kg of OTA results in lymphocyte depletion, medullary necrosis, cortical thinning, and increased apoptosis in immune organs [42]. We used 1 mg/kg of OTA for 21 days of continuous exposure and observed the condition of the chickens. In our study, chickens exposed to 1 mg/kg of OTA for 21 consecutive days exhibited characteristic symptoms including feather thinning, diarrhea, lethargy, retarded growth, and reduced spleen index. The OTA group demonstrated marked histopathological alterations including obscured red–white pulp demarcation, structurally compromised splenic nodules with decreased cellular density, and pathologically dilated sinusoids containing multiple hemorrhagic foci. Interestingly, AST supplementation effectively preserved splenic histoarchitecture, with tissue morphology comparable to the Control group and only minimal inflammatory cell infiltration observed. These findings confirm the successful establishment of our OTA-induced splenic injury model, enabling subsequent mechanistic investigations.

OTA has been demonstrated to compromise humoral immunity in chicks by significantly reducing specific antibody titers. This immunosuppressive effect leads to vaccination failure and increased susceptibility to secondary infections, including coccidiosis and Newcastle disease. [42,43]. Furthermore, this inhibition affects the breeder’s offspring. Progeny from OTA-exposed breeders exhibited significantly reduced spleen weights and markedly diminished serum immunoglobulin levels (IgA, IgG, and IgM) following maternal consumption of contaminated feed for 14–21 days [44]. Dietary supplementation with AST has been shown to alleviate thiacloprid (TCP)-induced hepatotoxicity and immunotoxicity through the attenuation of oxidative stress and suppression of inflammatory responses [45]. AST exhibits superior immunomodulatory properties, enhancing both the proliferation and functional capacity of immunoreactive cells [46,47]. Our immunoglobulin assay results corroborate these findings, demonstrating that OTA exposure significantly reduced serum IgM and IgG levels, while AST co-treatment effectively restored these immunoglobulin concentrations. These observations suggest that AST confers protection to immune organs, at least in part, through modulation of humoral immunity by maintaining IgM and IgG levels.

Oxidative stress exerts a biphasic effect on immune function: moderate levels enhance immune responses, while excessive oxidative stress leads to immunosuppression [48]. Free radicals produced by excessive oxidative stress can directly damage cell membranes and disrupt the normal function of immune cells [49]. Mitochondria are an essential source of ROS production and a major target of ROS invasion [50]. Excessive ROS accumulation triggers a vicious cycle by damaging mitochondrial DNA and respiratory chain proteins, which amplifies oxidative stress and ultimately induces apoptotic cell death [51]. We observed elevated levels of ROS and MDA in the spleen of chickens exposed to OTA, accompanied by a significant decrease in T-AOC activity. However, the addition of AST not only reduced ROS but also restored T-AOC activity and attenuated MDA accumulation. Moreover, oxidative stress impairs stem cell differentiation, inhibits the production of T and B cells, and compromises immune system efficiency, ultimately resulting in a decline in immune function [52,53]. Elevated ROS levels also trigger the excessive production of pro-inflammatory cytokines (IL-8, IL-6, and IL-1β) [54,55], which play pivotal roles in immune regulation [56]. Sustained splenic inflammation may lead to functional impairment and increased susceptibility to infectious diseases [57]. Our analysis revealed a significant upregulation of IL-8, IL-6, and IL-1β mRNA expression in OTA-exposed groups, correlating with pronounced splenic inflammation and impaired immune function. AST supplementation effectively suppressed these pro-inflammatory cytokine responses. These findings provide compelling evidence that OTA exposure induces both oxidative stress and inflammatory responses in splenic tissue. Furthermore, dietary AST administration demonstrated significant protective effects, attenuating OTA-induced oxidative damage and inflammatory cascades in spleens.

The PTEN/PI3K/AKT (Ser473) signaling pathway plays a pivotal role in regulating oxidative stress and apoptotic processes. We assay the expression of target genes and proteins related to the PTEN/PI3K/AKT (Ser473) signaling pathway and apoptosis. Phospho-AKT (Ser473) activation demonstrates consistent cytoprotective effects across diverse experimental models. Multiple studies have established its crucial role in cellular protection mechanisms: in sepsis-associated liver injury, monotropein (MON) attenuates oxidative stress, inflammation, and apoptosis by activating the AKT (Ser473)/GSK3β (Ser9)/Fyn/NRF2 signaling pathway [58]; fibroblast growth factor 21 (FGF-21) enhances bone regeneration by promoting bone mesenchymal stem cell (BMSC) differentiation and suppressing apoptosis through HGF-mediated PI3K/AKT (Ser473) pathway activation [59]; tanshinol borneol ester (DBZ) exerts neuroprotective effects by reducing microglial activation and oxidative stress via modulation of the AKT (Ser473)/GSK3β (Ser9)/Fyn pathway [60]. The PTEN/PI3K/AKT (Ser473) pathway is also associated with ROS. Elevated ROS levels activated PTEN, which in turn inhibited the PI3K/AKT (Ser473) signaling pathway [61]. OTA exposure induces excess ROS, upregulates PTEN expression, and inhibits the PI3K/AKT pathway [62,63]. Bcl-2 and Bax, downstream proteins of the PI3K/AKT pathway, are key indicators of apoptosis. Bax activates the apoptosis execution-phase enzyme caspase9, which subsequently regulates downstream caspase3 activity [64,65]. We observed that increased levels of ROS and PTEN protein expression in the OTA group inhibited the PI3K/AKT (Ser473) pathway, leading to a reduced Bcl-2/Bax ratio. In contrast, the AST + OTA group showed decreased ROS levels, downregulated PTEN expression, and enhanced PI3K/AKT (Ser473) pathway activation, resulting in an elevated Bcl-2/Bax ratio. Cleaved Caspase9 corresponds to activated Caspase9. Cleaved Caspase9 protein levels were significantly higher in OTA-treated spleens than in the Control group, and downstream Caspase3 levels were also elevated, indicating apoptosis. AST supplementation reduced Cleaved Caspase9 production. Further studies demonstrated that AST attenuates OTA toxicity by lowering ROS levels and PTEN expression, promoting PI3K/AKT (Ser473) pathway activation and thereby reducing apoptosis.

## 5. Conclusions

In conclusion, AST exerted protective effects against OTA-induced splenic dysfunction in chickens. AST increased immune organ indices and serum immunoglobulin (IgM and IgG) concentrations while alleviating apoptosis and inflammatory responses by mitigating OTA-induced oxidative stress and modulating the PTEN/PI3K/AKT (Ser473) pathway. This study provides a new theoretical basis and research direction for using AST to prevent and treat OTA-induced immunotoxicity.

## Figures and Tables

**Table 1:** List of gene primers for qPCR.

Name	Sense Strand/Sense Primer (5′–3′)	Antisense Strand/Antisense Primer (5′–3′)	Accession No.
PTEN	ACTCACTCTTGGCGAAGGAA	CTCCTGCTCCACCAACACTA	XM_015278701.2
PI3K	CTTCGGATGTTGCCTTACGG	GACACAGTAGCCAGCACAAG	NM_001004410.1
AKT	CCTTTTGTGGACCCTTCTGC	AGAAAATACCGTGGCCTCCA	NM_205055.1
Bcl-2	TTCAAGCGAAAACAGGGTGG	CTCTGAGCACATGGAAAGCC	NM_205339.2
Bax	CACCTTTGTCTCACCTGTGC	GATGGCAGTGATGAGCATGG	XM_015290060.2
Caspase3	TTGAAGCAGACAGTGGACCA	GTTCAAGTTTCCTGGCGTGT	NM_204725.1
IL-8	GGCTTGCTAGGGGAAATGA	AGCTGACTCTGACTAGGAAACTGT	NM_204608
IL-6	CAAGGTGACGGAGGAGGAC	TGGCGAGGAGGGATTTCT	NM_001277996
IL-1β	ACGTGGCAGCTTTTGAAGAT	GCGGTGGTTTTGTAACAGTG	XM_46931582
β-actin	CCCACACCCCTGTGATGAAA	TAGAACTTTGGGGGCGTTCG	NM_205518.1

## Data Availability

The data from this study are available on reasonable request to the corresponding authors.
